# A *Drosophila ex vivo* model of olfactory appetitive learning

**DOI:** 10.1038/s41598-017-17955-1

**Published:** 2017-12-18

**Authors:** Ema Suzuki-Sawano, Kohei Ueno, Shintaro Naganos, Yoshihiro Sawano, Junjiro Horiuchi, Minoru Saitoe

**Affiliations:** 1 Learning and Memory Project, Tokyo Metropolitan Institute of Medical Science, 2-1-6 Kamikitazawa Setagaya, Tokyo, 156–8506 Japan; 2 Department of Mathematics and Information Science, Tokyo Metropolitan University, 1-1 Minami Ohsawa, Hachioji, Tokyo, 192–0397 Japan

## Abstract

During olfactory appetitive learning, animals associate an odor, or conditioned stimulus (CS), with an unconditioned stimulus (US), often a sugar reward. This association induces feeding behavior, a conditioned response (CR), upon subsequent exposure to the CS. In this study, we developed a model of this behavior in isolated *Drosophila* brains. Artificial activation of neurons expressing the *Gr5a* sugar-responsive gustatory receptor (*Gr5a* GRNs) induces feeding behavior in starved flies. Consistent with this, we find that in dissected brains, activation of *Gr5a* GRNs induces Ca^2+^ transients in motor neurons, MN11 + 12, required for ingestion. Significantly, activation of *Gr5a* GRNs can substitute for presentation of sugar rewards during olfactory appetitive learning. Similarly, in dissected brains, coincident stimulation of *Gr5a* GRNs and the antennal lobe (AL), which processes olfactory information, results in increased Ca^2+^ influx into MN11 + 12 cells upon subsequent AL stimulation. Importantly, olfactory appetitive associations are not formed in satiated flies. Likewise, AL-evoked Ca^2+^ transients in MN11 + 12 are not produced in *ex vivo* brains from satiated flies. Our results suggest that a starved/satiated state is maintained in dissected brains, and that this *ex vivo* system will be useful for identification of neural networks involved in olfactory appetitive learning.

## Introduction

Animals can change their responses to sensory cues based on prior experiences. In conventional *Drosophila* olfactory conditioning^1^, flies are exposed simultaneously to an odor or conditioned stimulus (CS), and an unconditioned stimulus (US), consisting of a reward or punishment. Learning and memory of this association can be calculated at various times after training by measuring the percentage of flies displaying a conditioned response (CR), either approach to or avoidance of the CS^1–3^. Genetic analyses have identified various genes and structures involved in this behavior, and, more recently, *in vivo* functional imaging in living flies has shed light on activity of neural circuits associated with olfactory memory^4–8^. However, because it is difficult to keep anchored flies healthy for long periods of time under a microscope, in many studies, memory-associated changes in neural activities are obtained by comparing conditioned flies with unconditioned flies. Thus, it is still largely unknown how activity of a single individual circuit changes during formation, retention, and retrieval of a memory.

In aversive olfactory conditioning, CS and US information become associated in a brain structure known as the mushroom bodies (MBs). Previous *ex vivo* imaging studies demonstrated that input from projection neurons (PNs), which convey odor information from the antennal lobes (ALs) to the MBs, become associated with input from shock sensing pathways in the MBs, to produce long-term enhancement (LTE) of connectivity between PNs to MB Kenyon cells^9,10^. However, the relevant motor neurons required for aversive olfactory behaviors are unknown, as are the connections between MB Kenyon cells and these motor neurons. In appetitive olfactory conditioning, the CR is feeding behavior, which includes a proboscis extension response (PER) and pumping, required for ingestion^11^. Motor neurons involved in feeding behavior, including the MN11 + 12 motor neurons required for ingestion-associated pumping, have been identified. In this study, we examined whether activity of MN11 + 12 is altered in isolated dissected brains after simultaneous stimulation of the ALs and sugar gustatory receptor neurons (GRNs) that express gustatory receptor, *Gr5a* ^12–14^.

## Results

### Simultaneous odor and sugar presentation induces odor-associated PER

PER is an established assay for detecting appetitive olfactory memory in the honey bee *Apis* ^15^ and the fruit fly *Drosophila* ^11^. To measure appetitive olfactory memory using PER in anchored *Drosophila*, we starved flies for 20 hrs and then exposed them to 3-octanol, the odor used as the CS, while simultaneously exposing them to the US, 0.5 M sucrose. Because some immobilized starved flies displayed spontaneous proboscis extension even in the naïve state, we excluded these flies before conditioning (Supplemental Table S2).

**Figure 1:**
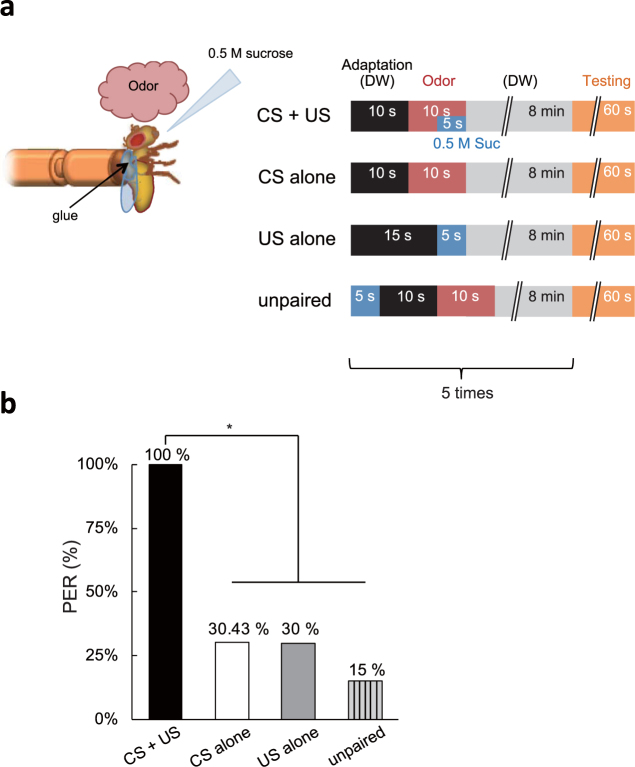
Olfactory appetitive learning in anchored *Drosophila*. (**a**) Conditioning protocol for olfactory appetitive learning. DW: distilled water, CS: conditioned stimulus, US: unconditioned stimulus. (**b**) Percentage of flies (*w* ^1118^(CS)) exhibiting proboscis extension response (PER) to the conditioned odor (3-octanol) after training. All flies in the CS + US conditioned group exhibited PER to the CS, whereas this percentage was significantly reduced in flies conditioned without sucrose (CS alone), without odor (US alone) or using the unpaired conditioning protocol. N = 20–27 and *P < 0.05 by chi-square test of independence.

Following an adaptation period with distilled water (DW), flies were exposed to the CS odor, 3-octanol, for 10 sec. During the last 5 sec of odor exposure, they were also presented with the US reward, 0.5 M sucrose (Fig. 1a). Appetitive conditioning consists of five training sessions with 8 min intervals between training sessions. 8 min after the 5th training session, we examined whether flies displayed PER during a 1 min exposure to the CS. We found that all conditioned flies displayed PER upon CS exposure. In contrast, flies trained with the CS alone, the US alone, or trained using an unpaired conditioning protocol displayed significantly lower PER frequencies (Fig. 1b). This indicates that our training protocol induces olfactory appetitive memory.

### Activation of *Gr5a* GRNs can replace sugar presentation during reward learning

**Figure 2:**
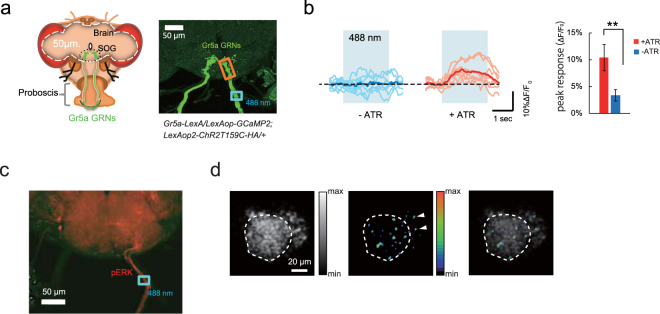
Blue light-dependent activation of *Gr5a* GRNs. (**a**) Stimulation of *Gr5a* GRNs. Left, A schematic illustration of a fly head and *Gr5a* GRNs. *Gr5a* GRNs project their axon terminals to the subesophageal ganglion (SOG). Right, The axon bundle of *Gr5a* GRNs (blue box) was stimulated using a 488 nm laser, and Ca^2+^ responses were recorded at the *Gr5a* GRN terminal area (orange box). (**b**) Ca^2+^ responses in *Gr5a* GRNs stimulated at 488 nm (light blue shaded areas indicate light stimulation). Blue traces, responses in brains from control flies (*w* ^1118^; *Gr5a-LexA::VP16/LexAop-GCaMP2; LexAop2-ChR2T159C-HA/*+) that were not fed ATR (−ATR). Red traces, responses in brains from flies fed ATR for 2 to 4 days before dissection (+ATR). Dark lines represent average responses. N = 8. The dashed line represents *F*_0_ in all imaging data. Right panel, peak responses from brains of flies fed ATR (+ATR) and not fed ATR (−ATR). **P < 0.01 by t-test. Error bars in all bar graphs represent standard errors of the means. (**c**) Increase in phosphorylated ERK (pERK) after photo-stimulation (blue box) of the *Gr5a* GRN axon bundle in a representative *w* ^1118^; *UAS-GCaMP3/Gr5a-LexA::VP16/;MB247-GAL4/LexAop2-ChR2T159C-HA* fly brain. (**d**) Ca^2+^ responses in the calyx of the MBs upon photo stimulation of *Gr5a* GRNs in a representative *w* ^1118^; *UAS-GCaMP3/Gr5a-LexA::VP16/;MB247-GAL4/LexAop2-ChR2T159C-HA* fly brain. The left panel shows a grayscale image of background GCaMP fluorescence in the calyx (circumscribed by white-dashed line), the middle panel shows the change in GCaMP fluorescence in pseudo color upon photostimulation of the ipsilateral *Gr5a* GRN, and the right panel shows both images merged. Arrowheads indicate fluorescence induced outside the calyx in the Kenyon cell soma.

*Gr5a* is a G-protein coupled receptor, expressed in gustatory receptor neurons (GRNs), that is required for the detection of the sugar, trehalose^12,14,16^. Previous studies have demonstrated that artificial activation of *Gr5a* expressing GRNs (*Gr5a* GRNs) induces PER^17,18^. Thus, we next examined whether artificial activation of *Gr5a* GRNs can replace sugar presentation during olfactory appetitive learning. We first developed a system to activate *Gr5a* GRNs by expressing photo-activated channelrhodopsin-2 (ChR2) and the Ca^2+^ indicator GCaMP2^19^ in these neurons using *Gr5a-LexA/LexAop-GCaMP2; LexAop2-ChR2T159C-HA/*+flies (Fig. 2a). We observed Ca^2+^ influx in *Gr5a* GRN axon terminal regions upon photo-stimulation of the *Gr5a*-GRN axon bundle in brains dissected from flies fed all trans retinal (+ATR) (Fig. 2b). ATR is required for channelrhodopsin-2 activity, and responses were not observed in brains from flies that were not fed ATR (−ATR) (Fig. 2b). Photo-stimulation of *Gr5a* GRNs from ATR-fed flies also increased phosphorylated ERK (pERK), a marker of neural activity, in *Gr5a* GRN axon bundles (Fig. 2c). In addition, we confirmed results from previous studies^17,18^, demonstrating that photo-stimulation of *Gr5a* GRNs induces PER in ATR-fed flies (unpublished observations). These data indicate that ChR2-expressing *Gr5a* GRNs can be activated by photo-stimulation, and activation induces appetitive responses.

A previous study demonstrated that sucrose feeding induces activation of dendritic claws in the calyx of the mushroom bodies (MBs)^20^. Consistent with this, we observed Ca^2+^ influx in the calyx of the MBs in response to photo-activation of *Gr5a* GRNs in dissected brains (Fig. 2d and Supplementary Fig. S1), indicating a functional connection between *Gr5a* GRNs and the MB neurons.

**Figure 3:**
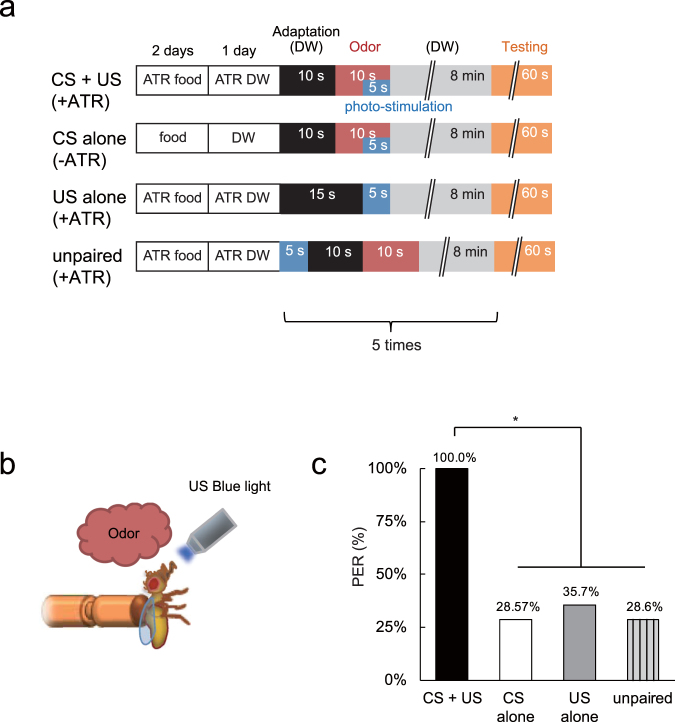
*Gr5a* GRN activation can replace sugar presentation during appetitive conditioning. (**a**) Experimental protocol. Flies in the +ATR group were fed ATR containing fly food for 2 days and ATR containing DW for one day before conditioning. Instead of a sucrose reward, *w* ^1118^; *UAS-ChR2T159C-mCherry; Gr5a-GAL4* transgenic flies were exposed to blue light during training. (**b**) Schematic diagram of the experimental setup. (**c**) The percentage of ATR fed flies that displayed PER to the CS after indicated conditioning protocols. In this case, the US refers to exposure to blue light. N = 10–21, *P < 0.05 by chi-square test of independence.

We next attempted to form appetitive associations in flies by pairing odor exposure with *Gr5a* GRNs activation. *UAS-ChR2T159C-mCherry; Gr5a-GAL4* transgenic flies were fed ATR for 3 days (+ATR) before being subjected to 5 training sessions (Fig. 3a), each consisting of a 10 sec odor exposure paired with a 5 sec exposure to blue light, followed by an 8 min inter trial interval (Fig. 3b). After conditioning, 100% of associatively conditioned (CS + US) flies displayed PER upon exposure to the CS odor, while a significantly lower fraction of flies trained with the CS alone, light exposure alone (US alone) or an unpaired conditioning protocol displayed PER upon CS exposure (Fig. 3c). Thus, artificial activation of *Gr5a* GRNs can replace sucrose presentation during reward learning.

### Coincident AL and *Gr5a* GRN stimulation alters motor neuron responses in dissected brains

**Figure 4:**
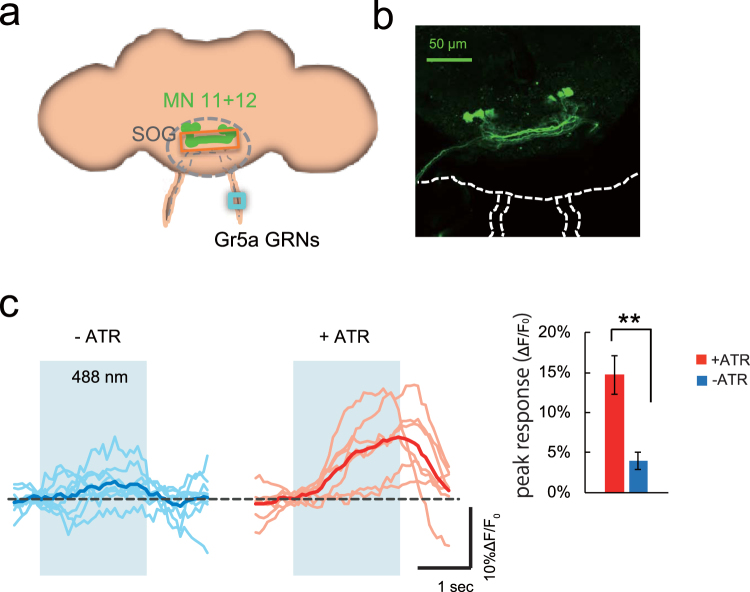
Activation of *Gr5a* GRNs induces Ca^2+^ responses in MN11 + 12. (**a**) A schematic of a fly brain indicating the locations of the SOG and MN11 + 12. The ROI for imaging is indicated by the orange box, and the site of laser light stimulation is indicated by the blue box. (**b**) GCaMP3 fluorescent image of MN11 + 12 in *w* ^1118^; *UAS-GCaMP3/Gr5a-LexA::VP16;MN11* + *12-GAL4/LexAop2-ChR2T159C-HA* transgenic flies. (**c**) Ca^2+^ activity in MN11 + 12 in −ATR (left panel) and +ATR fed flies (middle panel). Fly genotypes are the same as in (**b**). The peak responses in +ATR fed flies are significantly higher than in −ATR flies (right panel). N = 7 (+ATR) and N = 9 (−ATR). **P < 0.01 by t-test.

Ingestion in *Drosophila* requires activity of pump muscles in the proboscis, including muscles 11 and 12, which are innervated by motor neurons, MN11 + 12^21^. Inactivation of MN11 + 12 suppresses pumping, while activation induces pumping^21^. To determine whether stimulation of *Gr5a* GRNs affects MN11 + 12 activity, we next expressed ChR2 in *Gr5a* GRNs and GCaMP3 in MN11 + 12. When we stimulated *Gr5a* GRNs using a 488 nm laser, we observed significant Ca^2+^ activity increases in NM11 + 12 neurons in brains of ATR fed flies (Fig. 4c), indicating that increases in Ca^2+^ responses in MN11 + 12 may be a useful correlate for feeding behavior in dissected brains. Although MN11 + 12 neurons regulate ingestion pumping, we did not observe oscillatory rhythmic changes in their Ca^2+^ responses. This is likely due to the slow kinetics of GCaMP, since the sugar-induced GCaMP responses in MN11 + 12 neurons in living flies also failed to show rhythmic changes^21^. In addition to MN11 + 12 neurons, E-49 and NP0833 neurons are also involved PER^18,22^. However, the Ca^2+^ responses in these neurons during photo stimulation of *Gr5a* GRNs were less significant (Supplemental Fig. S2). Therefore, we focused on MN11 + 12 neurons in the following experiments.

**Figure 5:**
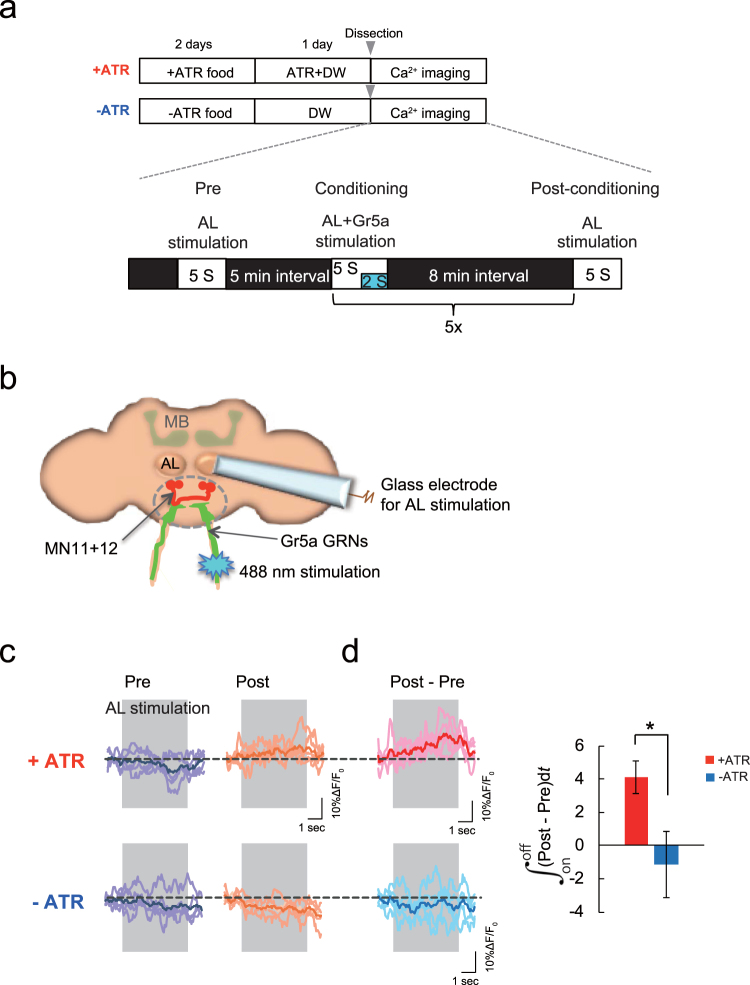
An *ex vivo* correlate of appetitive olfactory learning. (**a**) Protocol for *ex vivo* conditioning using AL and *Gr5a* GRN stimulation in *w* ^1118^; *UAS-GCaMP3/ Gr5a-LexA::VP16; MB247-GAL4/MN11* + *12-GAL4*, *LexAop2-ChR2T159C-HA* fly brains. (**b**) Diagram of the *ex vivo* system. A glass electrode is used for AL stimulation, 488 nm light is used for *Gr5a* GRN stimulation, and GCaMP activity is measured in MN11 + 12 neurons. (**c**) AL-evoked Ca^2+^ responses in MN 11 + 12 before (Pre) and after (Post) conditioning in brains from ATR fed (+ATR) and unfed (−ATR) flies. (**d**) Changes in AL-evoked Ca^2+^ responses after *ex vivo* conditioning (Post - Pre). Significantly increased responses were observed after conditioning in brains from ATR fed flies compared to responses in brains from flies not fed ATR. N = 5, *P < 0.05 by t-test.

We next examined whether coincident AL and *Gr5a* GRN stimulation alters AL-dependent Ca^2+^ responses in MN11 + 12 by electrically stimulating the AL and simultaneously activating *Gr5a* GRNs using 488 nm laser light (Fig. 5a,b). Since most single odors elicit > 50 Hz responses in PNs in the AL^23^, we stimulated the AL at 50 Hz for 5 s during concurrent *Gr5a* GRN activation. We applied 5 stimulation sessions with 8 min intervals between sessions. When we measured AL activity-dependent Ca^2+^ influx into MN11 + 12 at 8 min after the end of the last stimulation session, we found that Ca^2+^ influx was significantly increased (+ATR in Fig. 5d). This increase was not observed when *Gr5a* GRN activation was prevented by using brains from flies that were not fed ATR (−ATR in Fig. 5c,d). Furthermore, this increase was not observed when we stimulated *Gr5a* GRNs prior to AL stimulation (Supplemental Fig. S3b,c). Together, these results suggest that concurrent AL and *Gr5a* GRN stimulation induces plastic changes in the brain leading to increased MN11 + 12 responses to AL activity.

### MN11 + 12 responses are not enhanced in brains from satiated flies

**Figure 6:**
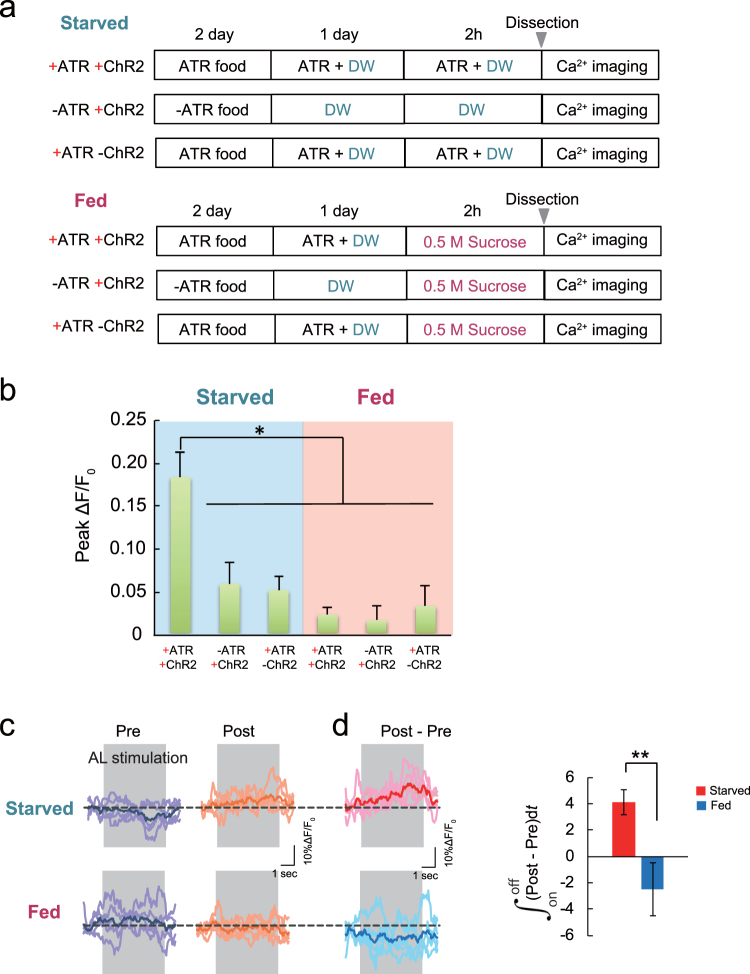
Differences in *ex vivo* plasticity in brains from starved versus fed flies. (**a**) Feeding and ATR treatment of *w* ^1118^; *UAS-GCaMP3/Gr5a-LexA::VP16; MB247-GAL4/MN11* + *12-GAL4*, *LexAop2-ChR2T159C-HA* and *w* ^1118^; *UAS-GCaMP3/Gr5a-LexA::VP16; MN11* + *12-GAL4/* + transgenic flies prior to dissection for *ex vivo* conditioning. Conditioning was carried out as described in Fig. 5a. (**b**) Photo-activation of *Gr5a* GRNs (+ATR + ChR2) significantly increased MN11 + 12 Ca^2+^ responses in brains dissected from starved flies, but not from fed flies. N = 6–9, *P < 0.05 by one-way ANOVA. (**c**) AL-evoked Ca^2+^ traces in MN11 + 12 before (Pre) and after (Post) *ex vivo* conditioning of brains from starved and fed flies. Data for starved flies is the same data shown in Fig. 5c. (**d**) Changes in AL-evoked MN11 + 12 responses after conditioning. *Ex vivo* conditioning significantly increased AL-evoked MN11 + 12 responses in brains from starved *w* ^1118^; *UAS-GCaMP3/Gr5a-LexA::VP16; MB247-GAL4/MN11* + *12-GAL4*, *LexAop2-ChR2T159C-HA* transgenic flies (adopted from +ATR, Fig. 5c) compared to responses in brains from fed flies. N = 5, *P < 0.05 by t-test.

Appetitive reward learning depends on the hunger state of the animal. Satiated flies are not motivated to feed, and consequently do not form appetitive associations. To test whether *Gr5a* GRNs-evoked MN11 + 12 activity in isolated brains is also affected by the hunger state of flies, we compared the peak of *Gr5a* GRN-evoked Ca^2+^ responses in MN11 + 12 in brains dissected from fed versus starved flies (Fig. 6a). We observed significant Ca^2+^ responses in brains isolated from calorie-starved flies expressing ChR2 and fed ATR (+ATR/+ChRs), compared to responses from brains from sugar-fed controls (Fig. 6b). Due to fluctuations in Ca^2+^ levels in the naïve state, we also observed slight Ca^2+^ responses in control brains dissected from starved flies expressing ChR2 but not fed with ATR (−ATR/ + ChR2) or from flies not expressing ChR2 but fed ATR (+ATR/−ChR2). However, these responses were not significantly different from responses from brains of fed flies. These results suggest that a motivational state induced by hunger is maintained in isolated brains.

We next examined whether associative responses in MN11 + 12 after coincident AL and *Gr5a* GRNs stimulation in dissected brains are also affected by the hunger state of donor flies. While we observed increased responses after paired stimulation in brains from starved flies (Starved, Fig. 6c adopted from Fig. 5c +ATR), we did not observe this increase in brains from satiated flies (Fed, Fig. 6c,d). Thus *Gr5a* GRN stimulation does not function as a US in dissected brains from satiated flies, further demonstrating the similarities between our *ex vivo* system and *in vivo* appetitive learning.

## Discussion

After olfactory appetitive reward conditioning, trained flies exhibit feeding behaviors when exposed to a reward-paired odor. In this study, we recapitulated the plasticity involved in this form of learning in *ex vivo*, dissected brains. We replaced odor presentation with electrical stimulation of the AL, since odor information is transmitted to the brain through the ALs. We also replaced sugar presentation with light stimulation of *Gr5a* GRNs since stimulation of these neurons both induces PER in naïve starved flies, and can replace sugar presentation in *in vivo* olfactory reward conditioning. As a correlate for feeding behavior, we measured Ca^2+^ responses in MN11 + 12. Using this system, we determined that AL stimulation does not induce Ca^2+^ responses in MN11 + 12 in naïve brains. However, *ex vivo* “training”, consisting of coincident AL and *Gr5a* GRN stimulation, induces plasticity in dissected brains, which can be measured as an increase in Ca^2+^ responses in MN11 + 12 upon AL stimulation. This increase did not occur in control brains where light-dependent *Gr5a* GRN stimulation was inhibited by withholding ATR prior to stimulation or *Gr5a* GRN stimulation was given prior to AL stimulation. Since *Gr5a* GRNs sense sweetness and MN11 + 12 is involved ingestion, our results suggest that plasticity associated with reward learning can be studied in dissected brain systems.

In *in vivo* preparations, immobilized starved flies sometimes display spontaneous proboscis extension even in the naïve state possibly due to various sensory inputs or internal conditions. Therefore, before training, we excluded flies displaying spontaneous proboscis extension in the naïve state. However, even after excluding these flies, a fraction of control flies trained by CS alone, US alone or unpaired conditioning still displayed PER after training. Again, we attribute this to variability present in living organisms. In contrast, in *ex vivo* preparations, MN11 + 12 did not show Ca^2+^ responses in the naïve state, suggesting that removal of sensory inputs to the brain reduces motor responses. Furthermore, AL stimuli did not evoke Ca^2+^ responses in MN11 + 12 unless dissected brains had been conditioned with coincident AL and *Gr5a* GRN stimulation. These differences between *in vivo* PER responses and *ex vivo* Ca^2+^ responses in MN11 + 12 suggest that *ex vivo* preparations may be useful for isolating specific neuronal associations from *in vivo* background activity.

Notably, in dissected brains derived from satiated flies, Ca^2+^ influx in MN11 + 12 did not increase upon *Gr5a* GRNs stimulation, or from AL stimulation after associative AL + *Gr5a* GRNs stimulation. This suggests that some aspects of the hunger/satiety state of living flies are preserved in dissected brains, and this state affects neuronal plasticity. A previous study suggests that activity of dNPF neurons correlates with motivational state during appetitive memory retrieval^24^. Thus, it is possible that activity of these neurons may preserve the hunger/satiety state in dissected brains. It will be of interest in the future to test whether activity of dNPF neurons influence Ca^2+^ influx in MN11 + 12 evoked by *Gr5a* GRNs stimulation, or by AL stimulation after associative AL and *Gr5a* GRNs pairing.

Sugar feeding has been shown to increase Ca^2+^ influx in the calyx of the MBs^20^. Similarly, we observed increased Ca^2+^ responses in this region upon *Gr5a* GRNs stimulations. These and other results suggest that neuronal activity from the ALs and *Gr5a* GRNs may become associated in the MBs. Indeed, previous *ex vivo* studies show that AL evoked Ca^2+^ responses in the vertical MB lobes become enhanced after associative stimulation of the ALs and the AFV (ascending fibers of ventral nerve cord), which transmit somatosensory information to the brain^4,9,10^. In the present study, however, we did not observe significant changes in AL-evoked Ca^2+^ responses in the vertical lobes after associative AL and *Gr5a* GRNs stimulation (data not shown), suggesting that olfactory appetitive learning may not require plasticity in the these lobes. Consistent with this idea, previous studies demonstrate that activation of dopaminergic neurons that innervate the horizontal lobes rather than vertical lobes are essential for reward learning^25,26^. Dopamine signaling plays an important role in neural plasticity in the MBs^9,10,27,28^. Although the location of the stimulatory microelectrode, which is placed adjacent to horizontal lobes, precluded precise recording of AL-evoked responses in the horizontal lobes, we suspect that plastic changes in AL-evoked Ca^2+^ responses in the horizontal lobes may occur after simultaneous AL and *Gr5a* GRNs stimulation. Thus, we speculate that the altered responses that we observe in MN11 + 12 may be caused by altered plasticity in the MBs. Since *ex vivo* preparations are more accessible for stimulation and recording than *in vivo* preparations, we believe that this system will provide novel insights into neural circuits and plasticity underlying olfactory appetitive learning.

## Methods

### Fly stocks and drug treatment

All fly stocks were raised on standard cornmeal medium at 25 C, and 60% humidity under a 12/12 h light/dark cycle. w(CS), containing the *w* ^1118^ allele in a Canton S background was used as the wild-type control^29^. 1–3 day old female flies were used for all experiments. For ATR feeding, 5 μL of 200 mM ATR (Sigma) dissolved in 95% EtOH was thoroughly mixed in 7 mL fly food as previously described^30^. For ATR feeding during starvation, ATR was dissolved at the same concentration in distilled water and applied to filter paper, which was then placed in fly vials.

### Transgenic lines

*LexAop2-ChR2T159C-HA* ^31^, and *UAS-ChR2T159C-mCherry* (provided by A. Nose, Univ. Tokyo) were used for photo-stimulation experiments. *UAS-GCaMP3* ^19^ (obtained from Bloomington Stock Center 32116 and 32237, Indiana Univ.), and *LexAop2-GCaMP2* ^9^ were used for measuring Ca^2+^ responses. *Gr5a-GAL4* ^32^ and *Gr5a-LexA::VP16* ^18^ (provided by K. Scott, UC Berkeley) were used to drive gene expression in *Gr5a* GRNs, and *NP1363* ^21^ (obtained from *Drosophila* Genetic Resource Center 112648) was used to drive expression in motor neurons for muscle 11 and 12 (*MN11* + *12-GAL4*) Each *E49-GAL4* ^18^ (provided by K. Scott) and *NP0833-GAL4* ^22^ (obtained from *Drosophila* Genetic Resource Center 103781) was used to drive expression in E49 motor neurons and feeding command neurons, respectively. *MB247* ^33^ was used for the MB-GAL4 driver. The precise genotypes of all flies used in the experiments are described in the Supplemental Table 1.

### Olfactory appetitive conditioning

For olfactory appetitive conditioning, we modified a protocol used in a previous study^11^. Briefly, flies were starved for one day before conditioning and individually glued on their backsides to the end of a toothpick. During a training session, mounted flies were first placed in a plastic petri dish (the adaptation chamber) containing filter paper dampened with distilled water for 10 seconds. They were then transferred to a second petri dish (the conditioning chamber) containing filter paper dampened with 3-octanol for 10 seconds. During the last 5 seconds in the conditioning chamber, filter paper saturated with 0.5 M sucrose was touched to the fly’s labellum. For optogenetic activation of *UAS-ChR2T159C-mCherry; Gr5a-GAL4* flies, instead of 0.5 M sucrose, flies were exposed for 5 sec to blue light (SR-01-B0023, Blue (470 nm) LUXEON LED - 48 lm @ 700 mA, Luxeon Star LEDs, Canada) at 350 mA to activate *Gr5a* GRNs^17,30^. The LED was placed in an aluminum foil half dome (diameter 1.5 cm) to direct light to the fly, and LED was placed 4 cm away from the fly. For experiments involving sucrose, flies were prevented from ingesting the sucrose by removing the sucrose saturated filter paper from the labellum immediately after touching. To form olfactory appetitive memory, flies were subjected to 5 training sessions with 8 min inter session intervals, and appetitive memory was measured as the percentage of flies that exhibited at least one PER during a 1 min exposure to 3-octanol.

Some starved and immobilized flies exhibited PER to various external stimuli including odors in the absence of training. We exclude flies exhibiting PER to 3-octanol before conditioning. The percentage of flies excluded is shown in Supplemental Table 2.

### Whole brain preparation

Brains were prepared for imaging as previously described^9^. Briefly, brains were dissected in ice cold Ca^2+^ free HL3 medium (70 mM NaCl, 115 mM sucrose, 5 mM KCl, 20 mM MgCl_2_, 10 mM NaHCO_3_, 5 mM trehalose, 5 mM Hepes, pH 7.3)^34^, and placed in a recording chamber filled with room temperature HL3 medium (the same recipe as above, containing 1.5 mM CaCl_2_).

### Imaging analysis

Fluorescent images were captured at 15 Hz using a two-photon laser scanning microscope (A1RMP, Nikon Corp., Tokyo, Japan), equipped with a 20x water-immersion lens (numerical aperture 0.5; Nikon Corp). To observe GCaMP fluorescence, we excited GCaMP at 950 nm and detected fluorescence through a 525–550 nm band pass filter. The *F* value was calculated for each pixel in the region of interest (ROI) using NIS-elements software (NIS-Elements, Nikon Corp.). 10 sequential frames before stimulus onset were averaged to obtain *F*_0_ values. *F* values and *F*_0_ values were used to calculated *ΔF/F*_0_. To optically stimulate *Gr5a* GRNs expressing ChR2, we used a 488 nm laser (incident laser power, 1.95 mW/cm^2^)^17,30^. Lasers for GCaMP excitation (950 nm) and for ChR2 stimulation (488 nm) were independently controlled using a resonant scanner to direct GCaMP stimulation and a galvano scanner to direct ChR2 stimulation. This allowed us to stimulate ChR2 at one ROI and measure GCaMP activity at a different location. To simulate odor input, we stimulated the AL using a glass electrode with an inner diameter of about 50 μm (the size of the minor axis of the AL). The electrode was connected to a stimulator (SEN-7103, Nihon Kohden) and an isolator (SS-104J, Nihon Kohden). We stimulated the AL with a train of 250 rectangle pulses (1 msec duration, 50 Hz for 5 sec)^9^. To determine the appropriate current amplitude for AL stimulation, we stimulated the AL (1 msec duration, 50 Hz for 5 sec) with gradually increasing currents and recorded Ca^2+^ responses in the MB. We identified the minimal amount of current needed to obtain stable Ca^2+^ responses and used this for stimulation. The current used was usually between 1–2 x the threshold current (the current at which responses are first detected in the Mbs). This method allowed us to stably stimulate the AL for several hours^9^. For Ca^2+^ response traces, each point represents the average ΔF/F_0_ from ten sequential frames. To quantify Ca^2+^ signals, we calculated the integral (area under the curve) of Post – Pre traces during 488 laser stimulation and compared values from flies fed ATR to those from flies not fed ATR.

### Immunostaining of pERK

*Gr5a* GRNs in *ex vivo* brains were stimulated using a 488 nm laser (1.95 mW/cm^2^) for 2 sec under a confocal microscope, and brains were fixed in 4% paraformaldehyde (PFA) for 2 to 3 h. After washing, fixed brains were incubated with rabbit monoclonal antibody against pERK (#4370 Phospho-p44/42 MAPK (Erk1/2), Cell signaling technology) at 1:500. After washing, brains were next incubated in secondary antibody, Alexa Fluor555 conjugated donkey anti-rabbit IgG (A31572, Life technologies) at 1:1000.

### Statistics

In imaging studies, we used the Student’s t-test for paired comparisons and the one-way ANOVA followed by the Tukey-Kramer method for multiple comparisons using StatView software (SAS Instruments). For PER experiments, we used the chi-square test of independence for multiple comparisons. *P < 0.05 and **P < 0.01 in all figures.

## Electronic supplementary material

Supplemental information
