# Supplementary material for: A Drosophila ex vivo model of olfactory appetitive learning
Source: Sci Rep. 2017 Dec 18;7:17725. doi: 10.1038/s41598-017-17955-1 (PMC5735177; doi:10.1038/s41598-017-17955-1)
Supplement: Supplementary file 1 — Supplemental information [file 41598_2017_17955_MOESM1_ESM.pdf]

Supplemental Information

A *Drosophila ex vivo* model of olfactory appetitive learning

Ema Suzuki-Sawano, Kohei Ueno, Shintaro Naganos, Yoshihiro Sawano, Junjiro Horiuchi, Minoru Saitoe

Supplemental Figure 1

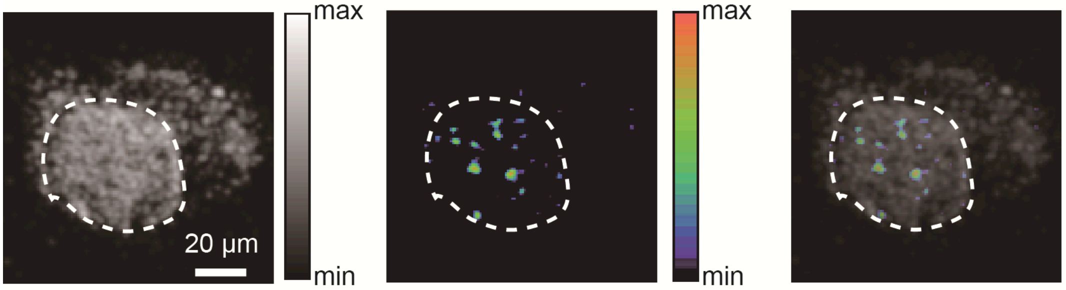

Supplemental Figure S1. Another example of Ca^{2+} responses in the calyx upon photo stimulation of *Gr5a* GRNs in a brain different from that of Fig. 2d. See the legend for Fig. 2d for details.

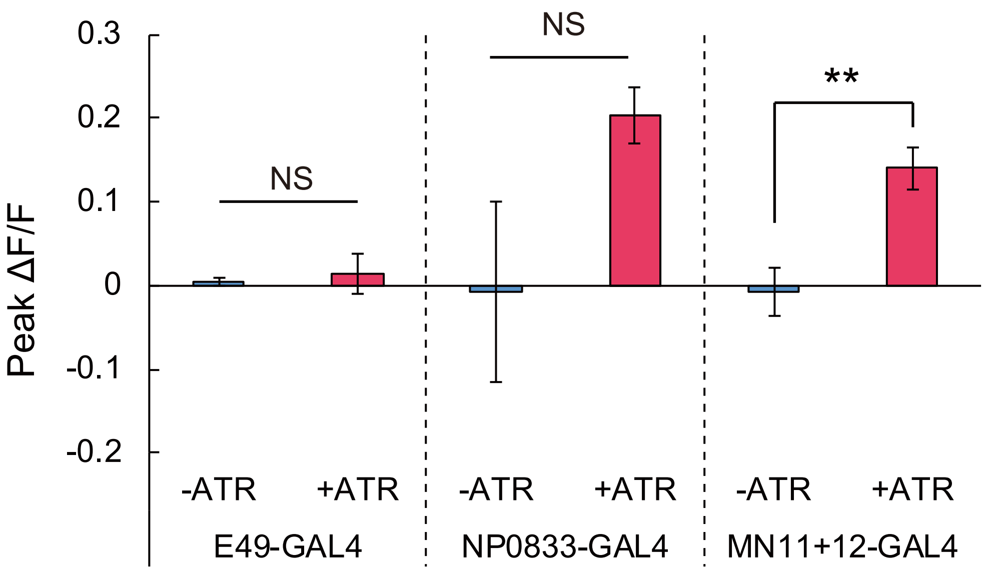

Supplemental Figure S2. The Ca^{2+} responses in the output neurons upon photo stimulation of *Gr5a* GRNs in three genotypes (*w*^{1118}*; E49-GAL4,/Gr5a-LexA::VP16;UAS-GCaMP3/LexAop2-ChR2T159C-HA,* *w*^{1118} *NP0833-GAL4/+;UAS-GCaMP3/Gr5a-LexA::VP16;LexAop2-ChR2T159C-HA/+*, and *w*^{1118}*; UAS-GCaMP3/Gr5a-LexA::VP16;MN11+12-GAL4/LexAop2-ChR2T159C-HA*). As in Figure 3, +ATR groups were fed ATR containing fly food for 2 days and ATR containing DW for one day before recording. -ATR flies were fed normal food for 2 days and DW without ATR for one day before recording. N=4-8, NS *P*>0.05, ***P* < 0.01 by t- test.

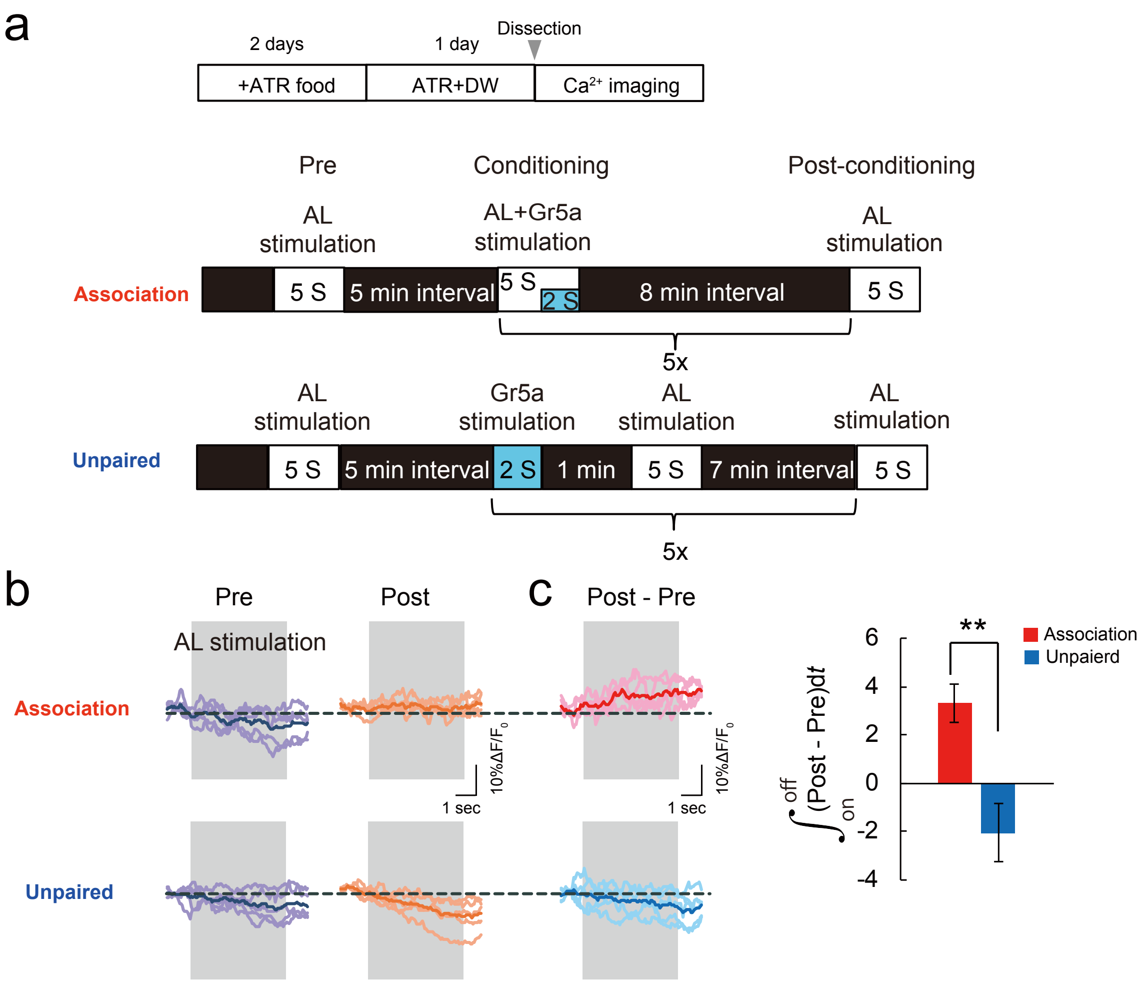

Supplemental Figure S3. Changes in AL-evoked MN11+12 responses after unpaired stimulation in *ex vivo* brains. Compared to associative stimulation, unpaired stimulation failed to increase AL-evoked MN11+12 responses in brains from w^{1118}; *UAS-GCaMP3/Gr5a-LexA::VP16; MB247-GAL4/MN11+12-GAL4, LexAop2-ChR2T159C-HA* transgenic flies. N = 6, $^{**}P < 0.01$ by t-test.

Figure	genotype
Fig. 1b	*w^{1118} (CS)*
Fig. 2a, b	*w^{1118}; Gr5a-LexA::VP16/LexAop-GCaMP2; LexAop2-ChR2T159C-HA/+*
Fig. 2c, d	*w^{1118}; UAS-GCaMP3/Gr5a-LexA::VP16/;MB247-GAL4/LexAop2-ChR2T159C-HA*
Fig. 3c	*w^{1118}; UAS-ChR2T159C-mCherry; Gr5a-GAL4*
Fig.4b, c	*w^{1118}; UAS-GCaMP3/Gr5a-LexA::VP16;MN11+12-GAL4/LexAop2-ChR2T159C-HA*
Fig. 5c, d	*w^{1118}; UAS-GCaMP3/Gr5a-LexA::VP16; MB247-GAL4/MN11+12-GAL4, LexAop2-ChR2T159C-HA*
Fig. 6b	*w^{1118}; UAS-GCaMP3/Gr5a-LexA::VP16; MB247-GAL4/MN11+12-GAL4, LexAop2-ChR2T159C-HA*
Fig. 6b	*w^{1118}; UAS-GCaMP3/Gr5a-LexA::VP16; MN11+12-GAL4/+*
Fig. 6c, d	*w^{1118}; UAS-GCaMP3/Gr5a-LexA::VP16; MB247-GAL4/MN11+12-GAL4, LexAop2-ChR2T159C-HA*
Supplemental Fig. 1	*w^{1118}; UAS-GCaMP3/Gr5a-LexA::VP16;MB247-GAL4/LexAop2-ChR2T159C-HA*
Supplemental Fig. 2	*w^{1118}; E49-GAL4,/Gr5a-LexA::VP16;UAS-GCaMP3/LexAop2-ChR2T159C-HA*
Supplemental Fig. 2	*w^{1118} NP0833-GAL4/+;UAS-GCaMP3/Gr5a-LexA::VP16;LexAop2-ChR2T159C-HA/+*
Supplemental Fig. 2	*w^{1118}; UAS-GCaMP3/Gr5a-LexA::VP16;MN11+12-GAL4/LexAop2-ChR2T159C-HA*
Supplemental Fig. 3b, c	*w^{1118}; UAS-GCaMP3/Gr5a-LexA::VP16; MB247-GAL4/MN11+12-GAL4, LexAop2-ChR2T159C-HA*

Supplementary Table S1. Fly genotypes used in all experiments.

Percentage of excluded flies

Fig. 1b	
CS + US	29 %
CS alone	36 %
US alone	41 %
unpaired	30 %

Fig. 3c	
CS + US	51 %
CS alone	62 %
US alone	31 %
unpaired	40 %

Supplemental Table S2. The percentage of excluded flies. Flies that exhibited PER to 3-octanol in the naïve state were not used in the following experiments.
